# SILDSO: Dynamic Switching Optimization Scheme for Solar Insecticidal Lamp Based on Multi-Pest Phototactic Rhythm

**DOI:** 10.3390/s25237332

**Published:** 2025-12-02

**Authors:** Heyang Yao, Lei Shu, Xing Yang, Kailiang Li, Miguel Martínez-García

**Affiliations:** 1 College of Engineering, Nanjing Agricultural University, Nanjing 210031, China; 2018812100@njau.edu.cn; 2 NAU-Lincoln Joint Research Center of Intelligent Engineering, Nanjing Agricultural University, Nanjing 210031, China; kailiang_li@njau.edu.cn; 3 School of Enqineering & Physical Sciences, University of Lincoln, Lincoln LN67TS, UK; 4 College of Intelligent Manufacturing, Anhui Science and Technology University, Chuzhou 233100, China; xingyang@ahstu.edu.cn; 5 Department of Aeronautical and Automotive Engineering, Loughborough University, Loughborough WC1E 6BT, UK; m.martinez-garcia@lboro.ac.uk

**Keywords:** solar insecticidal lamp, Internet of Things, energy management, intelligent switch scheme, multi-pest phototactic rhythm

## Abstract

Grain crops are regarded as fundamental to China’s agricultural production and food security. Effective control of nocturnal phototactic pests is essential for ensuring crop yields and achieving sustainable agricultural development. However, traditional solar insecticidal lamps often suffer from low energy utilization efficiency, dynamic switching control schemes, and poor adaptability in multi-pest coexistence scenarios. A multi-period intelligent switching control optimization scheme based on integrating a multi-pest phototactic rhythm is proposed, focusing on *Cnaphalocrocis medinalis* and *Chilo suppressalis* in rice fields. By considering the phototactic behavioral rhythm, energy consumption patterns, and residual energy levels, the proposed scheme dynamically optimizes the switching cycles of solar insecticidal lamps to maximize pest control effectiveness and energy efficiency. The rhythm modeling approach and dynamic adjustment mechanisms are employed to accurately align insecticidal working hours with varying pest activity patterns, thereby improving the pest control effectiveness of IoT-based solar insecticidal lamps. Simulation experiments demonstrate that, compared to traditional switching control schemes, the dynamic switching control scheme improves the average insecticidal rate by 17.7%, increases the effective insecticidal energy efficiency value by approximately 66.1%, and enhances the energy utilization rate by about 38.5%. The proposed dynamic switching control and intelligent energy management scheme not only improves the precision of pest control and energy utilization but also promotes the more efficient application of networked solar insecticidal lamps in smart agriculture. This work provides theoretical support and practical reference for intelligent pest control in complex agricultural environments, promoting the precision and sustainability of pest management practices.

## 1. Introduction

The production of grain crops serves as a critical foundation for ensuring national food security and maintaining social stability. As one of the largest producers and consumers of grain in the world, China relies heavily on the stable and high-yield production of major crops such as rice and wheat to safeguard food security [1]. However, pest infestations remain a major threat to crop yield and quality, causing annual economic losses amounting to hundreds of billions of yuan [2]. Among them, nocturnal phototactic pests, such as *Cnaphalocrocis medinalis* and *Chilo suppressalis* in rice fields [3], have emerged as significant biological stressors due to their complex activity patterns and the difficulties associated with their control [4,5]. The larvae of *Cnaphalocrocis medinalis* and *Chilo suppressalis* primarily feed on rice leaves and stems, causing structural damage that significantly hinders plant growth. As these larvae mature into adults, they oviposit near the crop fields, resulting in subsequent generations of larvae and recurring infestations. Therefore, in addition to targeting the larval stage, controlling adult insects is equally important. Effective suppression of adult populations can reduce oviposition rates and larval densities, thereby indirectly mitigating yield losses in rice cultivation.

**Figure 1:**
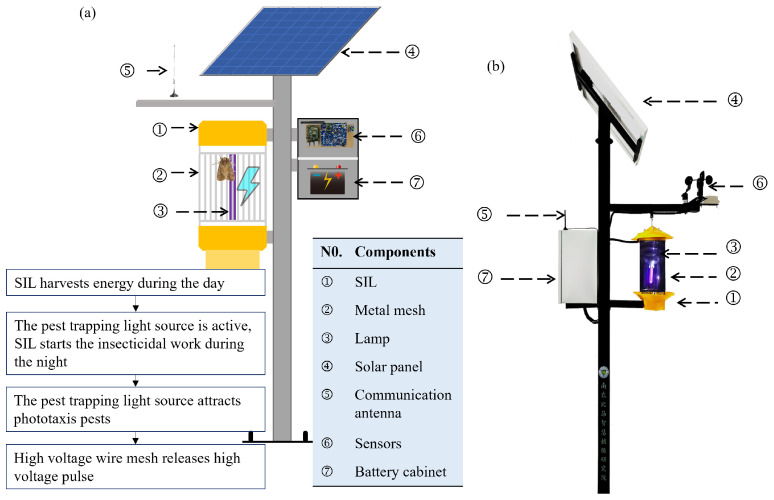
Modules of the SIL-IoT node. (**a**) System design diagram. (**b**) Physical diagram.

Traditional chemical pesticides, while effective in achieving rapid pest control, often result in unintended consequences with prolonged application [6]. These include the development of heightened pest resistance, environmental pollution, and compromised food quality and safety [7,8]. With the advancement of green and precision agriculture, physical pest control technologies have attracted increasing attention due to their environmentally friendly attributes and absence of pesticide residues [9,10]. In particular, solar insecticidal lamps (SILs), leveraging the phototactic behavior of pests, have shown promising application potential in grain crop pest management [11]. With the integration of Internet of Things (IoT) technology and intelligent algorithms providing new solutions for optimizing the control of SILs, IoT-enabled solar insecticidal lamp (SIL-IoT) systems have emerged as a new generation of physical pest control devices. The efficacy of precision control in these systems fundamentally relies on the effective utilization of pest phototactic behavior, with the selection of light source wavelength being a critical determinant. Major rice pests, including *Cnaphalocrocis medinalis* and *Chilo suppressalis* demonstrate significant spectral tropism toward specific wavebands, exhibiting peak sensitivity to UV-A light in the 365–400 nm regions [12,13,14]. Although multispectral light combinations or time-shifted spectral switching strategies present theoretical optimization potential for better synchronization with diverse pest activity rhythms, this study adopts the well-established 365–400 nm UV-A band as the model baseline due to insufficient quantitative efficacy curves. This wavelength range has been widely deployed in agricultural pest control and possesses substantial empirical validation. These systems utilize wavelength-specific light sources to attract phototactic pests, which are then neutralized by high-voltage pulse discharges (typically 8–10 kV). This technology offers dual benefits: (1) elimination of pesticide residues and (2) preservation of ecological balance. However, its efficacy is critically dependent on the precise control of the illumination time and energy management, a challenge that this paper aims to resolve. Figure 1 shows the system design diagram and the physical diagram. These SIL-IoT nodes primarily consist of five core components: (1) solar panels, responsible for converting light energy into electrical energy; (2) lead-acid battery packs for energy storage; (3) trap pest light sources to lure pests; (4) high-voltage pulse metal mesh (output voltage 3000–6000 V); and (5) IoT modules (integrated with environmental sensors and wireless communication modules). Their operational principle demonstrates distinct intelligent characteristics: during the day, energy is stored through photovoltaic conversion, while at night, based on pest activity data and environmental parameters acquired from the IoT platform, the high-voltage metal mesh is activated according to the phototactic rhythm of pests to execute the insecticidal operation mode.

Currently, most SIL-IoT nodes employ a fixed-period switching control scheme, which struggles to adapt to the time-varying characteristics of phototactic rhythm across different pest species [15]. Although some studies have attempted to use illumination intensity as a switching criterion, these approaches still exhibit notable limitations: firstly, they predominantly rely on single environmental variables and fail to comprehensively account for the relationship with pest phototactic behavior; secondly, there is a general lack of modeling and analysis of phototactic rhythm variations in multi-pest coexistence scenarios; thirdly, existing energy management strategies mainly focus on energy conservation without achieving multi-objective co-optimization between insecticidal efficacy and energy consumption. Furthermore, under energy-constrained conditions, current solutions often adopt forced shutdown strategies and lack dynamic priority scheduling mechanisms based on pest activity patterns, potentially leading to system failure during critical control periods. These shortcomings significantly restrict the adaptability, energy efficiency, and control effectiveness of SIL-IoT in complex farmland environments, particularly in scenarios with multiple co-existing pest species.

Therefore, to enhance the stability and control performance of SIL-IoT in real agricultural environments, it is imperative to overcome the limitations of existing fixed strategies and develop intelligent control methods that integrate multi-pest phototactic rhythm modeling with dynamic energy management. This paper explores innovative approaches in the following aspects: proposing an intelligent switching control architecture based on multi-pest behavior modeling, constructing a dynamic energy management mechanism that balances insecticidal effectiveness and energy consumption optimization, and introducing intelligent algorithms for online strategy optimization. This research aims to provide theoretical and technical support for achieving precise, green, and efficient operation of SIL-IoT.

### 1.1. Contributions

This work provides an intelligent switch scheme for the SIL, aiming to trap more pests with less energy. This approach improves the insecticidal effectiveness and energy efficient use of the SIL. Specifically, the main contributions of this paper are summarized in the following:

- A multi-pest rhythmic fusion model is proposed for the first time to characterize the nocturnal activity patterns of major rice pests (e.g., *Cnaphalocrocis medinalis* and *Chilo suppressalis*), overcoming the limitations of the conventional single-pest model. A multi-pest rhythm fusion modeling approach is developed to characterize the nocturnal activity patterns of multiple major rice pests.
- A novel multi-period dynamic switching scheme is developed based on a Particle Swarm Optimization (PSO) algorithm. This scheme dynamically adjusts the operation of the SIL-IoT in response to real-time pest activity and energy availability, enabling adaptive and precise control.
- An integrated control strategy is formulated to synergize pest management with energy optimization. Extensive simulation results demonstrate that the proposed strategy significantly improves the average pest mortality rate by 37.2% and energy utilization efficiency by 42.6% under high-energy conditions, compared to conventional fixed-time methods.

### 1.2. Organization

The remainder of this paper is organized as follows: Section 2 reviews the related work on SIL technologies, pest phototactic rhythm modeling, and intelligent optimization algorithms. In Section 3, the modeling of multi-pest phototactic rhythm is introduced, and a system model is formulated based on pest activity patterns. Section 4 presents the PSO-based multi-period intelligent switching optimization scheme for enhancing pest control effectiveness and energy utilization efficiency. Simulation results and performance analysis are provided in Section 5 to validate the proposed method.

## 2. Related Work

To advance energy management in the SIL-IoT, this section systematically reviews relevant research. We first summarize key advances in SIL-IoT technologies such as node deployment and fault diagnosis, analyzing their implications for energy management. We then examine switching control strategies for other outdoor IoT devices in smart agriculture, providing insights for developing efficient SIL-IoT energy management.

### 2.1. Progress in SIL-IoT Research Directions

The SIL-IoT, as a novel agricultural IoT technology [16], has achieved research advancements in multiple key technical areas, including node deployment schemes, fault diagnosis techniques, anti-electromagnetic interference methods, pest monitoring and localization technologies, and equipment maintenance schemes. To further investigate energy management methods for the SIL-IoT, it is essential to systematically review existing research directions and evaluate their potential impacts on energy management. As shown in Table 1, a comprehensive analysis of these research areas can provide a solid theoretical foundation and technical support for optimizing energy management solutions.

(1) Node Deployment schemes
  Research on node deployment has evolved from simple to complex scenarios. For intricate farmland structures, ref. [17] proposed an innovative zoning optimization method, transforming irregular deployment into a combinatorial optimization problem and employing boundary-independent and partition-independent algorithms to reduce costs. Crucially, optimized node deployment directly influences energy harvesting efficiency: rational spatial layouts maximize solar energy utilization while minimizing node count and communication overhead, forming the foundation for enhanced energy management.
(2) Fault Diagnosis Techniques
  Fault diagnosis research can be categorized into active and passive fault types, establishing a theoretical foundation for subsequent studies. The distributed diagnosis systems leverage local data exchange to cut communication loads. These advances ensure timely fault detection—preventing abnormal energy drain—while distributed architectures optimize energy use through reduced data transmission [18].
(3) Anti-Electromagnetic Interference
  Addressing high-voltage discharge’s impact on communication stability,  [19] quantified interference via a microprocessor trigger metric, while  [20] suppressed noise using audio signal compression, thereby boosting transmission accuracy. Stable communication prevents energy-intensive retransmissions, critical for field-deployed devices.
(4) Pest Monitoring and Localization
  From basic counting modules [21] to hardware-software fusion and variational mode decomposition models [22], precision pest tracking enables dynamic lamp operation adjustments, eliminating wasteful energy use during low-activity periods.
(5) Maintenance schemes
  Evolving from manual to crowd-sourced and drone-assisted anti-theft systems [23], these solutions sustain device reliability, indirectly supporting consistent energy management.

In conclusion, an increasing number of researchers are focusing their studies on the key technological development and application of SIL-IoT. The rapid evolution of agricultural intelligence has positioned SIL-IoT as a prominent research domain within agricultural IoT. Current studies predominantly operate under the fundamental assumption of “stable energy supply in SIL-IoT systems.” However, it is noteworthy that research on intelligent energy management for SIL-IoT remains relatively scarce. This research gap directly leads to two critical issues: (1) unstable pest control functionality caused by energy supply fluctuations in nodes and (2) compromised long-term system reliability. To address these challenges, this paper innovatively proposes a cross-scenario knowledge transfer approach, systematically integrating mature energy management methods from other agricultural IoT scenarios to develop an intelligent energy management solution tailored to SIL-IoT node characteristics.

### 2.2. Switching Scheduling for the Outdoor IoT Devices

With the continuous advancement of green and smart agriculture, the demand for efficient utilization of energy and resources in agriculture has become increasingly urgent. Energy management and intelligent switching control schemes have emerged as critical tools for achieving intelligent operation and energy-efficient scheduling of agricultural devices. These methods have been widely investigated and applied in scenarios such as smart irrigation, greenhouse automation, environmental sensing nodes, and solar-powered IoT devices, as shown in Table 2.

While the energy management strategies employed in outdoor smart equipment such as intelligent irrigation systems cannot be directly applied to the SIL-IoT, their dynamic scheduling methodologies and multi-objective optimization approaches under energy-constrained conditions provide significant methodological references for this study. These systems face common challenges, including unstable solar power supply, limited energy storage, and the need for multi-objective optimization, making their research findings highly valuable for reference.

In irrigation management, extensive research has been conducted on the development of IoT-based systems to achieve efficient energy and water utilization. In [24], an IoT-based smart irrigation system was proposed to precisely control the motor’ s on/off timing. This design reduces manual labor, optimizes the use of water and fertilizers, and ultimately improves crop yield. To further reduce deployment costs, a cost-effective irrigation module utilizing MQTT and HTTP protocols was introduced in [25], enabling real-time monitoring of crop status and efficient remote irrigation control. In [26], a solar-powered IoT-integrated irrigation system was introduced, utilizing remote switching to achieve both irrigation precision and energy savings. To enhance adaptability across varying field zones and crop types, a zoning-based irrigation scheme with fuzzy logic control was described in [27], ensuring optimal soil moisture levels through dynamic water distribution. As described in [28], historical data on maize water demand and soil moisture thresholds were used to design a smart irrigation timing algorithm, which achieved better efficiency in water use and ensured the responsiveness of the crops. In another study, a fuzzy logic controller was applied for real-time water adjustment, enhancing system intelligence and energy performance [29]. A sensor-driven energy switching method was established in [30], allowing autonomous state transitions in remote field devices based on real-time energy supply. In [31], a greenhouse power management system was constructed using solar panels and environmental sensors with scheduled switching; however, its response to dynamic changes in the environment or the state of the crop remained limited.

In contrast to irrigation and greenhouse energy control, relatively few studies have addressed energy-aware pest management. Most existing SIL systems adopt fixed insecticidal working time, which fail to account for the phototactic rhythm of the pest. As a result, excessive energy usage during inactive periods and insufficient pest mortality during peak periods are frequently reported [4]. Attempts to integrate phototactic behavioral rhythm into SIL control schemes have been made in [32], but these studies primarily focused on a single pest specie and exhibited limited adaptability or energy efficiency. Research on multi-pest phototactic rhythm fusion and intelligent schemes under energy constraints remains scarce.

In summary, although switch-based energy management schemes have demonstrated initial success in agricultural IoT, most existing SIL-IoT systems cannot jointly perceive and respond to pest phototactic rhythm and real-time energy states. In multi-pest coexistence scenarios under constrained energy availability, it is imperative to develop intelligent energy management systems that integrate pest phototactic rhythm, and multi-period adaptive control. Such systems are essential for enhancing both insecticidal effectiveness and energy utilization efficiency of SIL-IoT nodes.

## 3. System Model

Energy is fundamental for monitoring pest numbers, data transmission, and sensor device operations in the SIL-IoT. The core models in SIL-IoT include the mathematical model of pest phototactic rhythm, dynamic switching control, the energy residual and energy cost–control benefit co-optimization model. These models together constitute the SIL energy management models, aimed at optimizing the insecticidal working time of SIL, thereby enhancing pest control efficiency and energy utilization.

### 3.1. Assumptions and Definitions

To establish a dynamic switching control and intelligent energy management system based on mathematical models of multi-pest phototactic rhythm, this paper proposes the following fundamental assumptions:

**Assumption** **1.** *The SIL-IoT possesses intermittent insecticidal operation capabilities and can execute multiple switch cycles within a single night period. This functional characteristic forms the implementation foundation of the dynamic switching strategy proposed in this paper. Although this assumption imposes higher reliability requirements on power modules and switching components, its practical feasibility is fully supported by modern solid-state relay technology and durable power electronic devices, which are specifically designed for switching operations. It should be noted that the required switching frequency in our scheme remains relatively low (typically only a few cycles per night), which falls entirely within the normal operating range of commercially available components.*

**Assumption** **2.** *The energy consumed by each on/off switching cycle is negligible. The total energy consumption is assumed to be linearly correlated only with the duration of active insecticidal work. Assumption 2 enables the core dynamic switching strategy. Its validity is supported by both experimental evidence and industrial component specifications. As demonstrated in [33], the energy consumed by a single switching operation of the relay controlling the insecticidal lamp is negligible, measuring approximately $10^{3}\;\mu J$. This value is 4–5 orders of magnitude lower than the energy consumed by a 15 *W* device operating for just one hour. Therefore, disregarding this minimal energy cost represents a justifiable simplification under the principle of materiality for system-level energy modeling and does not affect the core conclusions of this study.*

**Assumption** **3.** *During the predefined operational period, the SIL-IoT system remains fully reliable, and no unexpected shutdowns occur due to hardware or system failures. Since this paper addresses energy management through a dynamic start-stop control strategy, it is necessary to decouple the model from hardware reliability issues to simplify the modeling approach. Temporarily excluding hardware reliability variables allows the research focus to remain concentrated. This assumption establishes a foundation for subsequent research, while in practical applications, system reliability can be further enhanced through redundancy design.*

**Assumption** **4.** *Environmental variables during nighttime operation—such as temperature, humidity, and ambient light—are assumed to remain relatively stable and do not significantly affect pest phototactic behavior or the insecticidal performance of the system. Although extreme weather events may temporarily violate this assumption’s conditions, it maintains sufficient empirical foundation and engineering rationality in typical agricultural environments, ensuring the model’s validity and reliability in the vast majority of scenarios.*

To facilitate a clearer understanding of the proposed intelligent switching mechanism for the SIL-IoT, in addition to the terminology introduced in Section 4, the following definitions are also introduced in this section:

**Definition** **1.** *Switching Command: A switching command refers to a complete on/off cycle, wherein the SIL-IoT system is turned on and subsequently off once during nighttime operation.*

**Definition** **2.** *Energy Efficiency: Energy efficiency is defined as the effectiveness of the SIL-IoT in terms of the number of pests eliminated per unit of energy consumed, expressed as the ratio of eliminated pest count to total energy consumption.*

### 3.2. Mathematical Model of Pest Phototactic Rhythm

**Figure 2:**
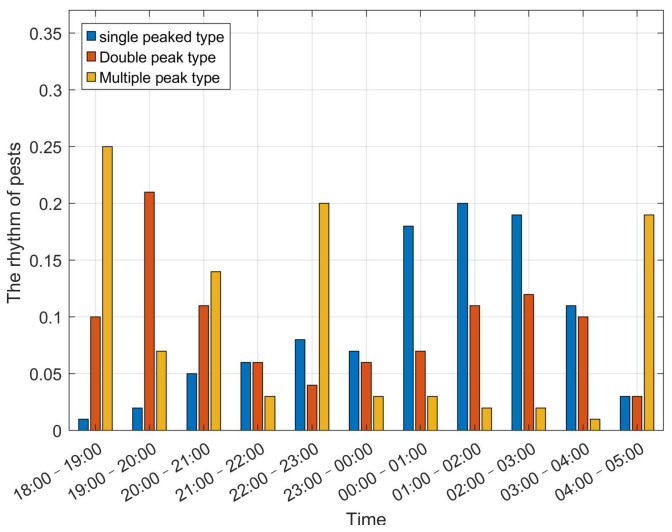
The distribution types of the phototactic rhythm of pests.

Nocturnal pests have developed a directed behavioral response to light sources through evolution, an ecological adaptation phenomenon referred to as phototactic rhythm [4]. This phototactic rhythm reflects the periodic behavioral responses of insects to light stimuli, which are closely linked to their internal biological clocks, photoperiod adaptation mechanisms, and diurnal activity patterns. According to existing research, pest phototactic rhythm patterns can generally be classified into three major categories: single-peak type [34,35,36], double-peak type [37], and multimodal-peak type [35], as shown in Figure 2. The y-axis in Figure 2 represents the proportion of pests attracted to the light trap per hour relative to the total nightly catch, which is a unitless ratio. Recently, the integration of SIL with pest phototactic behavior has progressively developed into a critical approach to agricultural pest monitoring and integrated management. Pest population dynamics are regulated by multiple factors, including seasonal variations, geographical characteristics, crop types, and environmental conditions such as temperature, humidity, precipitation, and wind direction. SIL-IoT systems are typically deployed during peak pest occurrence periods—from late spring to autumn. Taking eastern China as an example, the highest pest activity generally occurs between May and October.

Rice, as a major grain crop in China, is highly vulnerable to pests such as the rice leaf roller (*Cnaphalocrocis medinalis*), stem borers (e.g., *Chilo suppressalis*) and *Mythimna seperata* during its growth cycle [16]. Among these, the rice leaf roller and the striped stem borer are particularly destructive, with population increases leading to significant yield losses. The larvae of *Cnaphalocrocis medinalis* typically infest rice from the tillering to heading stages. They roll leaves into cylindrical structures using silk and feed on the mesophyll, damaging leaf tissue, impairing photosynthesis, and ultimately stunting plant growth. Meanwhile, *Chilo suppressalis* larvae bore into rice stems during the jointing to milk stages, feeding internally and disrupting vascular tissues, which results in dead hearts, white heads, and lodging—severely reducing grain yield.

As these larvae develop into adults, they lay eggs in the fields, exacerbating the damage caused by subsequent generations. Therefore, controlling both larval and adult stages is critical. Effective suppression of adults can reduce egg laying and larval density, thereby mitigating the impact on rice production.

In managing *Cnaphalocrocis medinalis* and *Chilo suppressalis*, physical control methods represent an environmentally sustainable scheme, particularly suitable for green and organic farming systems. This approach leverages the phototactic behavior of adult pests, using specific wavelengths of light (e.g., ultraviolet) to attract them, followed by high-voltage metal mesh to eliminate the insects with pulsed currents. This method not only reduces reliance on chemical pesticides but also supports sustainable rice cultivation.

To further enhance the pest control efficacy and energy utilization efficiency of SIL-IoT, this paper proposes an intelligent switching control scheme based on the phototactic rhythm of the two aforementioned major rice pests, aiming to support coordinated multi-pest management in monoculture rice systems. For the division of insecticidal time intervals, this paper adopts the same method as described in [32], clearly segmenting energy allocation into an energy supply period (06:00–19:00) and an energy consumption period (19:00–06:00 the next day). Since solar panels are the sole energy source of the device, when the lead-acid battery has insufficient stored energy, the system must rely on solar charging the following day to maintain operation. Therefore, the trap pest light source should be activated during periods of high pest activity at night, thereby simultaneously improving energy utilization efficiency and insecticidal effectiveness.

Given that the device operates exclusively at night, this paper defines the continuous period from the activation to deactivation of the trap pest light source as the insecticidal operation duration, denoted as $t_{m}$. This duration corresponds to the uninterrupted time interval of nightly lamp operation. The 11-h nocturnal period is divided into 11 one-hour intervals. For example, $t_{m}=1$ corresponds to 19:00–20:00, $t_{m}=11$ corresponds to 05:00–06:00, and other values of $t_{m}$ represent the respective consecutive hourly segments accordingly.

This paper aims to apply and optimize a dynamic switching control scheme for SIL-IoT in rice fields. Based on mathematical models of the phototactic rhythm of two major pest species—*Cnaphalocrocis medinalis* and *Chilo suppressalis*—the proposed scheme enables intelligent regulation of the operational timing and duration of SIL-IoT during nighttime. Following the modeling approach proposed in [16,32], the phototactic rhythm of *Cnaphalocrocis medinalis* is described by the following mathematical expression:

(1) $$\begin{array}{c} p_{1}\left(t_{m}\right)=-4.607\times 10^{-5}\times t_{m}^{5}+0.001\times t_{m}^{4}\\ -0.017\times t_{m}^{3}+0.109\times t_{m}^{2}-0.39\times t_{m}+0.645 \end{array}$$

where $t_{m}$ represents the temporal distribution of pest activity during the night. When $t_{m}=1$, this corresponds to the time period from 19:00 to 20:00.

The modeling equation for the phototactic rhythm of the *Chilo suppressalis* is as follows:

(2) $$\begin{array}{c} p_{2}\left(t_{m}\right)=0.286\times \exp\left(-\frac{{\left(t_{m}-2.911\right)}^{2}}{2\times 1.009^{2}}\right)+0.059\times \exp\left(-\frac{{\left(t_{m}-8.685\right)}^{2}}{2\times 2.026^{2}}\right) \end{array}$$

where $t_{m}$ represents the temporal distribution of pest activity during the night. When $t_{m}=1$, this corresponds to the time period from 19:00 to 20:00. Based on the nocturnal activity patterns of two major rice field pests, this paper establishes a time-dependent one-dimensional phototactic rhythm model, as shown in Equation (3), which quantitatively characterizes the nighttime phototactic behaviors of the pests.

(3) $$p_{\mathrm{static}\;}\left(t_{m}\right)=p_{1}\left(t_{m}\right)+p_{2}\left(t_{m}\right)$$

For more accurate modeling of the nocturnal phototactic rhythm (19:00-6:00 next day) of *Cnaphalocrocis medinalis* and *Chilo suppressalis* in rice fields, this paper introduces ecological disturbance factors [38] based on the static model. The mathematical model for the dynamic phototactic rhythm of these two major rice pests is presented in Equation (4):

(4) $$f\left(t_{m}\right)=P_{\mathrm{static}\;}\left(t_{m}\right)+\xi \left(t_{m}\right)$$

where $\xi \left(t_{m}\right)$ represents Gaussian white noise, modeling random disturbances in the environment, as shown in Equation (5):

(5) $$\xi \left(t_{m}\right)=\sigma \cdot \epsilon_{m}$$

Among them, σ=0.05 represents the standard deviation. $\epsilon_{i}∼N\left(0,1\right)$.

This dynamic model serves as the basis for an adaptive scheme of the SIL-IoT, enabling energy-aware pest control schemes aligned with the pests’ nocturnal activity.

### 3.3. Dynamic Switching Control Model

Based on the nocturnal phototactic rhythm of two major pests in the mid-tillering stage of rice—*Cnaphalocrocis medinalis* and *Chilo suppressalis*—this paper sets the effective operational period of the SIL-IoT from 19:00 to 06:00 the following day. To accurately model the state of the trap pest light source during nighttime operation, a switching control method is proposed to characterize its switch status across different time intervals. Using an equally spaced discretization approach, the 11-h operational period is divided into 11 standard time units ($t_{m}=1,2,\ldots ,11$), each corresponding to 60 min. The method constructs a binary state sequence $\alpha_{t_{m}}\in {\left\{0,1\right\}}^{11\times 1}$ to represent the switching behavior over discretized time intervals $\alpha_{t_{m}}=\left[\alpha_{1},\alpha_{2},\ldots \alpha_{10},\alpha_{11}\right]$. Here,

$\alpha_{t_{m}}$ denotes the operational state of the SIL-IoT at the $t_{m}$ time unit, where

$\alpha_{t_{m}}=0$ indicates that the trap pest light source is off (i.e., insecticidal operation is suspended);

$\alpha_{t_{m}}=1$ indicates that the light source is on (i.e., insecticidal operation is active).

Through this binary representation, the operational state of the SIL-IoT can be unambiguously described for each time unit. The formulation is expressed as follows:

(6) $$\begin{array}{c} \alpha_{t_{m}}=\left[\alpha_{1},\alpha_{2},\ldots \alpha_{10},\alpha_{11}\right]\\ \alpha_{t_{m}}\in \left\{0,1\right\},\;\forall t_{m}\in \left\{1,2,\ldots 10,11\right\} \end{array}$$

The nighttime period from 19:00 to 06:00 the following day is divided into 11 equal-length time intervals. The switch state of each interval directly affects the total insecticidal operation duration and energy consumption. For example, a sequence such as $\alpha_{t_{m}}=\left[0,1,1,1,1,1,1,1,0,1,1\right]$ indicates that the SIL-IoT is off during 19:00–20:00, on from 20:00 to 03:00, off during 03:00–04:00, and on again from 04:00 to 06:00. Therefore, the total insecticidal operation time of the SIL-IoT refers to the cumulative duration during which the trap pest light source remains active at night, denoted as $T_{on}$. This can be calculated by summing the state values (i.e., counting the number of intervals where the state equals 1) in the switching state sequence. Similarly, the total time during which the SIL-IoT is off and insecticidal operation is suspended, denoted as $T_{off}$, can be obtained by subtracting $T_{on}$ from the total number of time intervals.

Each interval’s state change directly influences the calculation of the total nightly operational duration, thereby enabling precise evaluation of the operational efficiency of the SIL-IoT across different periods. Accordingly, the total insecticidal operation time $T_{on}$, can be expressed as

(7) $$T_{on}=\sum_{t_{m}=1}^{11}\alpha_{t_{m}}$$

The duration during which the SIL-IoT ceases insecticidal operation by deactivating the trap pest light source is termed the non-operational period $T_{off}$ and can be expressed as

(8) $$T_{off}=11-T_{on}$$

The Equation (8) can be further expressed as

(9) $$T_{off}=11-\sum_{t_{m}=1}^{11}\alpha_{t_{m}}$$

### 3.4. Residual Energy Model for Multi-Component Energy Consumption

The activation of the trap pest light source in the SIL-IoT generates an instantaneous current surge. However, due to its extremely short duration, the energy consumption associated with this transient current is negligible. Therefore, when the SIL-IoT operates with only one or a few switching cycles per night, the energy cost of such current surges can be practically ignored.

Based on this, the energy consumption model of the SIL-IoT in this paper does not account for the transient switching actions of turning the light source on or off. Instead, it focuses on the following primary energy consumption components: (1) energy consumed by the trap pest light source; (2) energy consumed by the high-voltage metal mesh; (3) energy consumed by high-voltage insecticidal pulses; (4) energy consumed by the IoT module. The trap pest light source used in this paper is consistent with the equipment described in [32]: a 15 W blacklight lamp, which exhibits high efficacy in attracting pests. When no pests come into contact with the high-voltage metal mesh, it remains in a static, non-discharging state with a static power consumption of 1.8 W.

Unlike [32], this paper focuses specifically on the control of two major pests in rice fields—*Cnaphalocrocis medinalis* and *Chilo suppressalis*—as representative grain crop threats. Accordingly, the energy consumption of high-voltage insecticidal pulses is considered only for eliminating these two target pest species.

Through detailed analysis of these energy consumption sources, this paper aims to optimize the energy allocation scheme of the SIL-IoT, thereby further improving both pest control efficiency and energy utilization efficiency for the two primary pests in rice cultivation.

Hence, the energy consumption of SIL-IoT $E_{c}^{t_{m}}$ in $t_{m}$ time slot is mainly (1) the energy consumption of lamp $E_{lc}^{t_{m}}$, (2) the energy consumption of metal mesh $E_{mc}^{t_{m}}$, (3) the energy consumption of insecticidal $E_{pc}^{t_{m}}$ and (4) the energy consumption of IoT communication transmission $E_{tc}^{t_{m}}$. Thus, the energy consumption of SIL-IoT $E_{c}^{t_{m}}$ can be modeled as

(10) $$\begin{array}{c} E_{c}^{t_{m}}=E_{lc}^{t_{m}}+E_{mc}^{t_{m}}+E_{pc}^{t_{m}}+E_{tc}^{t_{m}} \end{array}$$

The residual energy model adopts the method described in [32], where the remaining energy within a specific time interval $t_{m}$ is primarily determined by two factors: the initial residual energy $E_{0}$ after the solar panel charges the lead acid battery during the day and the energy consumed $E_{c}^{t_{m}}$ during night insecticidal operation. Therefore, the residual energy $E_{r}^{t_{m}}$ of the SIL-IoT is modeled as shown in Equation (11):

(11) $$E_{r}^{t_{m}}=E_{0}-E_{c}^{t_{m}}$$

Therefore, the residual energy $E_{r}^{t_{m}}$ of the SIL-IoT can be further expressed by Equation (12) as

(12) $$E_{r}^{t_{m}}=E_{0}-\left(E_{lc}^{t_{m}}+E_{mc}^{t_{m}}+E_{pc}^{t_{m}}+E_{tc}^{t_{m}}\right)$$

The initial State of Charge of the SIL-IoT $SOC_{0}$ is defined as the energy status of the 48 V lead-acid battery when the trap pest light source is activated at night. It can be expressed as the ratio of the energy capacity $E_{0}$ of the SIL-IoT at the moment of light source activation to the total capacity $E_{a}$ of the 48 V lead-acid battery. The initial SOC of the SIL-IoT can be calculated as

(13) $$\begin{array}{c} SOC_{0}=\frac{E_{0}}{E_{a}} \end{array}$$

### 3.5. Energy Cost–Control Benefit Co-Optimization Model

The SIL-IoT primarily rely on energy stored in lead-acid batteries during the daytime to perform insecticidal operations at night. However, limited energy capacity may lead to operational failures at critical moments due to power deficiency, resulting in missed optimal control opportunities. Additionally, the high initial investment and ongoing maintenance costs of the SIL-IoT necessitate a balanced consideration of both economic efficiency and pest control effectiveness in their deployment.

Since the primary function of the SIL-IoT is to eliminate major crop pests, the previous research [32] focused on maximizing insecticidal efficacy for a single dominant pest species without addressing cost-related issues. However, in practice, SIL-based pest management must account not only for the insecticidal rate but also for operational costs to avoid missing key intervention windows caused by energy depletion. Given the relatively high market price of SIL-IoT systems, deployment in rice fields requires consideration of both pest density and overall cost-effectiveness. The costs of SIL-IoT systems include initial equipment acquisition, long-term maintenance, and energy consumption. Due to these substantial costs, SIL-IoT systems are typically deployed in a sparse configuration.

To improve the effectiveness of SIL-based pest management, optimizing the operational schedule of a single SIL is essential before addressing collaborative scheduling of multiple lamps within a region. Therefore, this paper focuses on energy-efficient management of the insecticidal operation time through intelligent switching of the trap pest light source for a single SIL. As the core of energy optimization lies in rational allocation of operational time, which directly affects energy consumption, the cost analysis in this paper primarily emphasizes battery energy cost.

To better evaluate the benefits of the SIL-IoT under varying conditions, a comprehensive benefit model is established to quantify the trade-off between pest control effectiveness and energy cost. This model uses the difference between the effective energy utilization rate and the crop loss rate due to pests as its core metric. Within a given period, a higher number of pests eliminated by the SIL corresponds to lower crop loss and thus reduced economic damage in rice fields. Therefore, the crop loss rate caused by pests can be indirectly reflected by the insecticidal rate of the SIL. Specifically, a higher insecticidal rate reduces the pest population, thereby decreasing the crop loss rate. Through mathematical modeling or experimental validation, the insecticidal rate can be translated into the probability of pest elimination, enabling quantification of its impact on field loss rate.

This model allows for a holistic assessment of the integrated benefits of the SIL-IoT in pest control, providing a scientific basis for optimizing dynamic switching control schemes. The comprehensive benefit model is given by Equation (14):

(14) $$W^{t_{m}}=f\left(t_{m}\right)-\frac{E_{c}^{t_{m}}}{E_{0}}$$

Among them, $E_{0}$ represents the energy capacity of the SIL-IoT when the trap pest light source is activated, $E_{a}$ denotes the total energy consumption of the SIL-IoT, and $f\left(t_{m}\right)$ indicates the activity level of pests during the nighttime interval $t_{m}$. By constructing this comprehensive benefit model, a systematic and quantitative evaluation of the multi-faceted benefits of the SIL-IoT in agricultural production can be achieved. This approach not only facilitates scientific decision-making and promotes widespread application but also provides a theoretical foundation for optimizing resource allocation, thereby further advancing sustainable agricultural development and green production practices.

### 3.6. Parameter Transformation

To address the distinct nocturnal activity patterns of two major pests in rice crops, this paper proposes dividing the nighttime operational period of the SIL-IoT into 11 intervals. Each interval can be assigned one of two states: activating the trap pest light source for pest control or deactivating it to suspend operations. Thus, the operational state of each SIL-IoT becomes a decision variable to be optimized.

By introducing a scheme involving multiple activations and deactivations of the light source, the insecticidal operation time of the SIL-IoT can be controlled more flexibly and precisely, adapting to the nighttime activity rhythm of the target pests across different intervals. Through rational allocation of switch states for each time interval, this optimization approach not only enhances pest control efficacy but also maximizes energy utilization efficiency at various stages.

Consequently, this paper focuses on transforming the problem of repeatedly switching the light source into a complex scheduling optimization problem, aiming to design a more efficient operational schedule for the SIL-IoT to control *Cnaphalocrocis medinalis* and *Chilo suppressalis*. Specifically, the working state of the SIL-IoT will be adjusted based on peak and off-peak periods of pest activity. By establishing a mathematical model of pest phototactic rhythm, the switch state of each time interval will be optimized to maximize insecticidal efficacy during high-activity periods while reducing energy consumption during low-activity periods.

Furthermore, this paper explores how IoT technology can be utilized to monitor and adjust the operational states of the SIL-IoT in real time, adapting to dynamic pest activity patterns and environmental conditions. Ultimately, this optimization method aims to achieve a more efficient and intelligent pest control solution, providing robust support for agricultural production.

### 3.7. Problem Statement

Generally speaking, improving energy efficiency requires reasonable utilization of the residual energy in the SIL-IoT. This challenge is particularly critical for pest control using SIL-IoT systems. The core research problem of such systems often focuses on major pests like *Cnaphalocrocis medinalis* and *Chilo suppressalis* in rice crops. The aim is to optimize operational scheduling to achieve an optimal balance between insecticidal efficacy and energy utilization.

Addressing the complex characteristics of inconsistent nocturnal activity patterns among multiple pests, particularly the overlapping and divergent peak activity periods of these two species, an optimization model for dynamic switching control of the SIL-IoT is constructed.

Due to the high energy consumption of the trap pest light source, the operational duration of the SIL-IoT is positively correlated with energy consumption, which directly affects the economic feasibility of its insecticidal operations. Therefore, improving insecticidal efficiency within limited time intervals not only enhances energy utilization but also significantly reduces the overall operational cost of the SIL-IoT system.

Studies have shown that selectively activating the trap pest light source during high-frequency pest activity periods at night can markedly enhance both insecticidal effectiveness and energy utilization efficiency. Based on this, the optimization objective in this paper is defined as maximizing insecticidal efficacy while minimizing energy consumption, integrated into a comprehensive utility function. Specifically, the objective function can be expressed by the following formula:

(15) $$\;\mathrm{Object}:\;\max\alpha_{t_{m}}f\left(t_{m}\right)$$

(16) $$\min E\left(t_{m}\right)$$

Here, $f(t_{m})$ denotes the distribution density of pests during the $t_{m}$ time interval at night. $\alpha_{t_{m}}$ represents the operational state of the SIL-IoT during the $t_{m}$ time interval. $\alpha_{t_{m}}=\left[\alpha_{1},\alpha_{2},\ldots \alpha_{10},\alpha_{11}\right]$ is a [111] binary vector where $\alpha_{t_{m}}\in \left\{0,1\right\}$. Specifically, $\alpha_{t_{m}}=0$ indicates that the SIL-IoT is in the off state (deactivating the trap pest light source and suspending insecticidal operation) during interval $t_{m}$, while $\alpha_{t_{m}}=1$ indicates that the SIL-IoT is in the on state (activating the trap pest light source and performing pest trapping and elimination). $E(t_{m})$ denotes the proportion of energy consumption during interval $t_{m}$.

Since the energy consumption of the SIL-IoT is related to the timing and duration of its operation, and the periods with high pest abundance do not necessarily correspond to the highest insecticidal efficiency, a multi-objective optimization problem is transformed into a single-objective optimization problem to simultaneously address insecticidal efficacy and energy cost while reducing computational complexity. Specifically, by introducing weighting coefficients, the two objectives—maximizing insecticidal effectiveness and minimizing energy consumption—are integrated into a comprehensive objective function. Therefore, the objective function is formulated as shown in Equation (17):

(17) $$F\left(t_{m}\right)=\alpha_{t_{m}}\left(\gamma f\left(t_{m}\right)-\beta E\left(t_{m}\right)\right)$$

To make the optimization objective more intuitive and avoid biases introduced by subjective weight selection, this paper normalizes both insecticidal effectiveness and energy consumption and sets all weighting coefficients to 1. γ=1,β=1. Thus, the objective function can be transformed into the form shown in Equation (18).

(18) $$\begin{array}{c} F\left(t_{m}\right)=\alpha_{t_{m}}\left(f\left(t_{m}\right)-\frac{E_{c}^{t_{m}}}{E_{0}}\right) \end{array}$$

(19) $$\begin{array}{c} \;\mathrm{Object}\;:\;\max\;F\left(t_{m}\right) \end{array}$$

Through the comprehensive benefit model, Equation (18) can be further transformed into Equation (20).

(20) $$\begin{array}{c} \;\mathrm{Object}\;:\;\max\;F\left(t_{m}\right)=\alpha_{t_{m}}\times W^{t_{m}} \end{array}$$

Here, $F\left(t_{m}\right)$ denotes the fitness function and optimization objective modeled in this paper. $E_{0}$ represents the energy capacity of the SIL-IoT when the insect-attracting light source is activated and $E_{c}^{t_{m}}$ indicates the total energy consumption of the SIL. This function simultaneously captures the trade-off between insecticidal efficacy and energy consumption, thereby simplifying the optimization process and improving computational efficiency.

### 3.8. Dynamic Switching Control Constraints

The design of a dynamic switching control and intelligent energy management scheme for the SIL-IoT, based on pest phototactic rhythm, must holistically integrate multiple constraints including energy supply, available battery capacity, and insecticidal efficacy. To ensure feasibility and effectiveness of the optimized operation scheme, this section specifies the following three key categories of constraints:

#### 3.8.1. Load Energy Balance

To maintain the energy balance of the SIL-IoT in rice fields, the load energy balance constraint must be satisfied. While the research in [32] primarily focused on energy consumption within individual time intervals, this subsection introduces an additional daily energy constraint. Specifically, the total daily energy consumption of the system must not exceed its energy budget, meaning it should remain within the integrated limits of battery capacity and the charging capability of the solar panels. This constraint is designed to prevent system operational failures or battery depletion due to excessive energy consumption. Its mathematical formulation is given by Equation (21):

(21) $$\begin{array}{c} E_{r}⩾\alpha_{t_{m}}E_{c}^{t_{m}} \end{array}$$

where $E_{r}$ denotes the residual energy of the SIL-IoT during the nighttime; $E_{c}^{t_{m}}$ represents the energy consumption of the SIL-IoT during time interval $t_{m}$; and $\alpha_{t_{m}}$ indicates the operational state of the SIL-IoT at time interval $t_{m}$, where $\alpha_{t_{m}}\in \left\{0,1\right\}$. Specifically, s(t)=0 indicates that the SIL-IoT is in the off state (deactivating the insect-attracting light source and suspending insecticidal operation) during interval $t_{m}$, while s(t)=1 indicates that the SIL-IoT is in the on state (activating the light source and performing pest trapping and elimination).

#### 3.8.2. Battery Available Capacity Constraint

To prevent over-charging/discharging of the lead-acid battery and extend its service life, a minimum SOC threshold must be defined to avoid deep discharge caused by excessively low battery capacity [39]. Specifically, when the remaining battery capacity of the SIL-IoT falls below 10% in any time interval (SOC < 10%), the system suspends insecticidal operation to prevent deep discharge [40]. Therefore, the dynamic switching control and intelligent energy management scheme of the SIL are primarily influenced by the initial residual energy, energy consumption, and residual energy during operation. The available capacity constraint of the lead-acid battery can be expressed as

(22) $$\begin{array}{c} SOC_{0}⩾10\% \end{array}$$

(23) $$\begin{array}{c} SOC_{t_{m}}⩾10\% \end{array}$$

#### 3.8.3. Insecticidal Efficacy Constraint

To ensure the effectiveness of the SIL-IoT across different time intervals and to guarantee overall pest control performance, the nightly insecticidal rate must not fall below 10%. This constraint is designed to ensure that, while optimizing the operational duration, the system remains capable of effectively responding to pest activity and achieving the desired control efficacy. Its mathematical expression is given by

(24) $$\begin{array}{c} \alpha_{t_{m}}f\left(t_{m}\right)⩾10\% \end{array}$$

Here, $f(t_{m})$ denotes the distribution density of pests during the $t_{m}$ time interval at night; $\alpha_{t_{m}}$ represents the operational state of the SIL-IoT during the $t_{m}$ time interval, where $\alpha_{t_{m}}\in \left\{0,1\right\}$.

## 4. Proposed Scheme

Multi-Peak Intelligent Switching Control Scheme for SIL-IoT: addressing the technical challenges associated with controlling multiple pests such as *Cnaphalocrocis medinalis* and *Chilo suppressalis* during the mid-tillering stage of rice crops, this paper proposes a multi-peak intelligent switching control scheme (hereinafter referred to as the “multi-peak control scheme”) for an SIL-IoT, based on modeling the nocturnal phototactic rhythm of multiple pests. By integrating the phototactic behavioral rhythm of these two pests, a multi-peak switching control model is constructed. Leveraging networked control technology, the scheme enables intelligent switching control of the SIL-IoT, aiming to simultaneously enhance pest control efficacy and energy utilization efficiency.

The proposed multi-peak control scheme employs a PSO algorithm to establish a multi-objective optimization control system for the SIL-IoT. By comprehensively analyzing key parameters such as pest phototactic rhythm, operational duration, initial residual energy, and comprehensive benefits, the system dynamically adjusts the timing and duration of insecticidal operations to achieve efficient pest control and optimal energy usage.

As a swarm intelligence optimization algorithm, PSO mimics the social behavior of bird flocks or fish schools to search for optimal solutions. Serving as the core optimization engine of the system, PSO exhibits strong alignment with the control requirements of SIL-IoT, which is reflected in three synergistic mechanisms:

- Generalization Capability: In response to the nonlinear relationship between SIL operational parameters and pest activity, the gradient-free optimization of PSO effectively overcomes the strict differentiability requirements of traditional PID control. Through swarm intelligence search mechanisms, the system handles optimization problems involving discontinuities and non-smooth regions.
- Global Search Ability: The parallel search mechanism enables simultaneous exploration of multiple regions in the solution space, significantly reducing the risk of premature convergence.
- Engineering Applicability: With concise parameter configuration (e.g., inertia weight and learning factors) and an adaptive inertia weight adjustment mechanism, the algorithm demonstrates excellent convergence stability.

**Figure 3:**
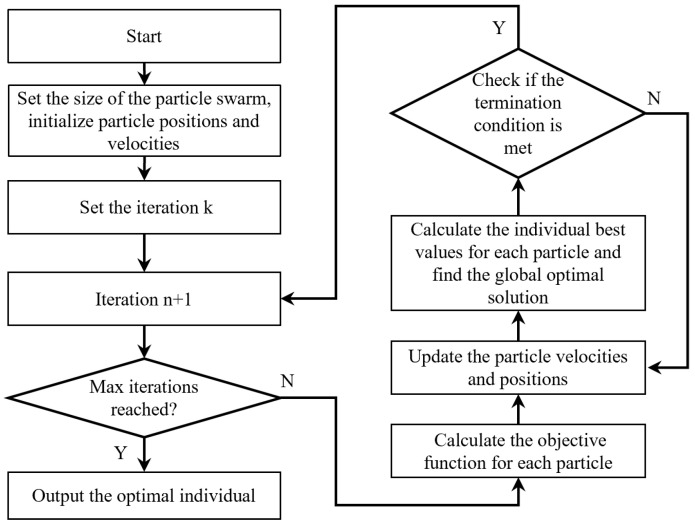
The flowchart of particle swarm optimization algorithm.

As shown in Figure 3, the implementation of the PSO algorithm involves four key steps: (1) Particle Encoding: binary encoding of the switch modes across 11 time units from 19:00 to 06:00; (2) Fitness Function Calculation: weighted evaluation based on pest control efficacy and energy consumption; (3) Velocity-Position Update: search direction adjustment via dynamic update equations; (4) Optimal Solution Output: application of a binary PSO algorithm to derive the optimal dynamic switching control schedule for insecticidal operation.

In the multi-peak intelligent switching control scheme, the PSO algorithm is primarily employed to address the following multi-objective optimization problem: under limited energy constraints, maximize both the number of pests captured and energy utilization efficiency, while satisfying constraints related to energy consumption, lead-acid battery available capacity, insecticidal effectiveness, and operational duration.

**Figure 4:**
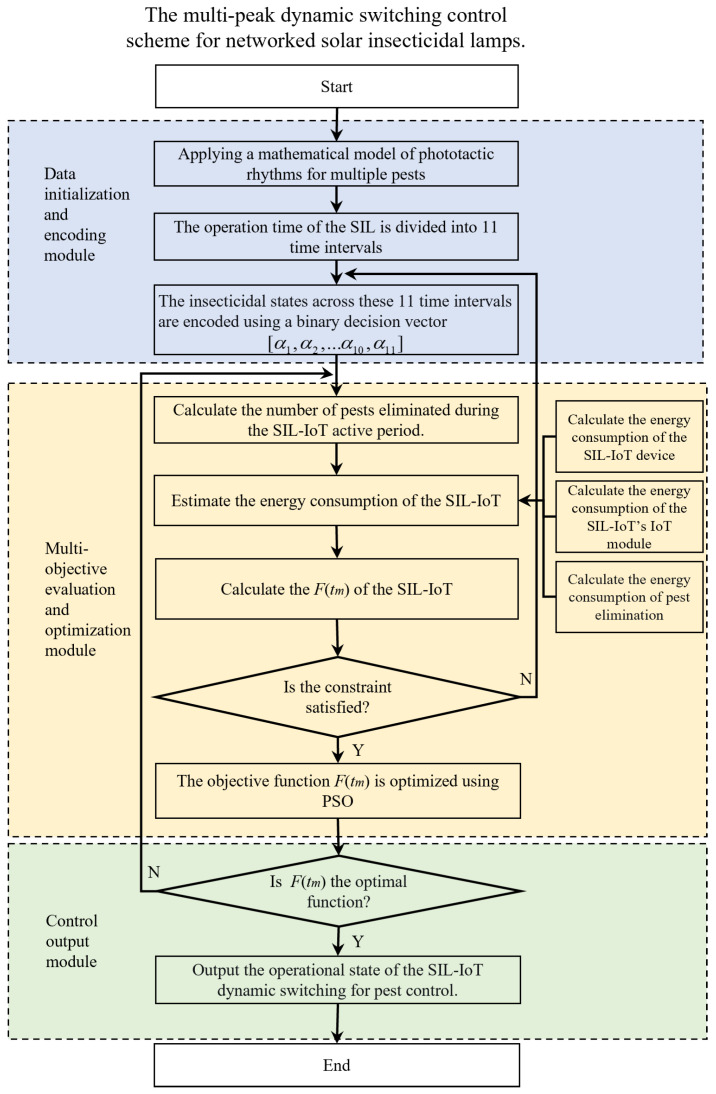
The flowchart of the multi-mode intelligent switch control scheme for networked solar insecticidal lamp.

As shown in Figure 4, the proposed scheme consists of three core modules: (1) Data initialization and encoding module, (2) Multi-objective evaluation and optimization module, and (3) Control output module.

First, all necessary parameters and settings are initialized based on mathematical models of the phototactic rhythm of multiple major pest species, providing a foundation for subsequent scheme selection and testing. Next, the number of pests eliminated and the energy consumption are calculated according to the insecticidal characteristics of the SIL-IoT, thereby establishing a multi-objective function. Finally, the decision vector representing the operational time states is iteratively adjusted using the PSO optimization algorithm until the objective function converges to an optimum, at which point the optimal insecticidal operation schedule is output.

Specifically, this paper divides a 24 h day into two distinct periods: an energy supply period during daytime (06:00–19:00) and an energy consumption period at night (19:00–06:00 the next day), using the hour as the fundamental time unit. Along this timeline, the operational state of the SIL-IoT is defined. Since pest phototactic behavior occurs exclusively at night, the SIL remains in the off state—deactivating the trap pest light source and suspending insecticidal operation—during the daytime energy supply period. Throughout this time, the solar panels charge the lead-acid battery to accumulate energy. In contrast, during the nighttime energy consumption period, each hourly interval offers two possible operational states: activating or deactivating the light source.

First, particle encoding is applied to the nightly insecticidal time intervals. The switch mode across the 11 nighttime intervals (from 19:00 to 06:00, with one hour per interval) is denoted as $\alpha_{t_{m}}=\left[\alpha_{1},\alpha_{2},\ldots \alpha_{10},\alpha_{11}\right]$, $\alpha_{t_{m}}\in \left\{0,1\right\}$. To regulate the switching time of the SIL, the problem is transformed into an optimization task.

Second, each particle’s position corresponds to the switch mode for each hour of the night. The position vector of a particle, denoted as $X_{id}=\left[x_{i1},x_{i2},x_{i3},\ldots ,x_{id}\right]$, represents a candidate solution, while its velocity vector, $V_{id}=\left[v_{i1},v_{i2},\ldots v_{i10},v_{i11}\right]$, indicates the search direction and step size. For particle *i* in dimension *d* at generation k+1, the velocity update follows Equation (25):

(25) $$\begin{array}{c} v_{id}^{k+1}=\omega v_{id}^{k}+c_{1}r_{1}\left(p_{\mathrm{best},id}-x_{id}^{k}\right)+c_{2}r_{2}\left(g_{\mathrm{best},d}-x_{id}^{k}\right) \end{array}$$

Here, ω denotes the inertia weight (typically ranging from 0.4 to 0.9), which controls the influence of historical velocity, thereby preserving the particle’s previous direction of motion and avoiding abrupt changes; *k* indicates the current iteration number of the PSO algorithm. $v_{id}^{k}$ represents the velocity of the particle *i* in dimension *d* at iteration *k*; $c_{1}$ and $c_{2}$ denote the learning factors (usually set as $c_{1}=c_{2}=2$ ), which represent the weights assigned to the individual best solution and the global best solution, respectively. $r_{1}$ and $r_{2}$ are random numbers uniformly distributed in the interval [0, 1], primarily simulating environmental uncertainties or equipment response errors; $p_{\mathrm{best}\;,id}$ denotes the historical best position of particle *i*; $g_{\mathrm{best}\;,d}$ represents the historical best position of the swarm in dimension *d* (global best); $x_{id}^{k}$ indicates the position of particle *i* in dimension *d* at iteration *k*.

The update formula for the position $x_{id}^{k}+1$ of particle *i* in dimension *d* at iteration k+1 is given by Equation (26):

(26) $$x_{id}^{k+1}=x_{id}^{k}+v_{id}^{(}k+1)$$

where $x_{id}^{k}$ denotes the position of particle *i* in dimension *d* at the *k* iteration; $v_{id}^{\left(k+1\right)}$ represents the updated velocity of particle *i* in dimension *d* at the *k* iteration. If $x_{id}^{k+1}$ exceeds the predefined boundaries, it is clamped to the nearest boundary value or reflected into the feasible domain.

Pseudocode of the Multi-Peak Intelligent Switching Control Scheme for the SIL-IoT is shown in Algorithm 1.

This scheme utilizes the PSO algorithm to intelligently regulate the activation and deactivation of the insect-attracting light source in the SIL-IoT based on peak pest activity periods. By doing so, it significantly improves energy utilization efficiency while ensuring effective pest control. During nighttime intervals with high pest activity, the SIL activates its light source to perform insecticidal functions, thereby maximizing control efficacy. Through this dynamic switching control scheme, the energy efficiency of the SIL system is enhanced, and the control effectiveness against multiple pest species is optimized. In summary, this paper systematically constructs a cooperative control and energy optimization model for the SIL-IoT. First, a mathematical model is established based on the patterns of pest phototactic rhythm curves. Subsequently, these mathematical models are integrated with an optimization algorithm. Ultimately, this method outputs the optimal operation time and duration for the SIL-IoT, not only enhancing pest control efficiency but also optimizing energy utilization efficiency.
**Algorithm 1** Pseudocode of the Multi-Peak Dynamic Switching Control Scheme for SIL-IoT**Require:** $t_{m}$, $f(t_{m})$, $SOC_{0}$ **Ensure:** $\alpha_{t_{m}}$, *T*, Nq, $SOC_{t_{m}}$, Best $f(t_{m})$  1:Create initial population  2:**for** $\forall \alpha_{i}\in \left[0,1\right]$ **do**  3:    $\alpha_{t_{m}}=\left[\alpha_{1},\alpha_{2},\ldots ,\alpha_{11}\right]$  4:    $T_{\mathrm{on}}\leftarrow \sum_{i=1}^{11}\alpha_{i}$  5:    $T_{\mathrm{off}}\leftarrow 11-T_{\mathrm{on}}$  6:    $X_{i}\leftarrow \left[x_{i1},x_{i2},\ldots ,x_{id}\right]$  7:    $V_{i}\leftarrow \left[v_{i1},v_{i2},\ldots ,v_{id}\right]$  8:    **if** $T_{\mathrm{on}}>0$ **and** $SOC_{0}>10\%$ **then**  9:        Statistical analysis of pest phototactic rhythm10:        $f(t_{m})\leftarrow$ according to the Equations (1)–(5)11:        Calculate energy consumption12:        $E_{i}^{k-1}\leftarrow$ according to the Equations (6)–(13)13:        $F\left(t_{m}\right)\leftarrow \alpha_{i}\left(\gamma f\left(t_{m}\right)-\beta E\left(t_{m}\right)\right)$14:    **end if**15:    $X_{i}^{k+1}\leftarrow X_{i}^{k}+V_{i}^{k+1}$ {according to the Equations (21)–(26)}16:    **while** $E_{i}^{k-1}\geq E_{\mathrm{threshold}}$ **do**17:        $SOC_{i}\leftarrow SOC_{0}-SOC_{i-1}$18:        **if** $SOC_{i}>10\%$ **then**19:            $F\left(t_{m}\right)\leftarrow \alpha_{i}W^{k-1}$20:            InsecticidalRate ← Calculate insecticidal rate21:            OperationTime ← Calculate operation time22:            $\alpha_{t_{m}}\leftarrow$ Find optimal switching state {according to the Equations (14)–(20)}23:        **end if**24:    **end while**25:**end for**26: 27:**return** $\alpha_{t_{m}}$, *T*, Best $F(t_{m})$

## 5. Simulation

This section evaluates the performance of the proposed dynamic switching control and intelligent energy management system through extensive simulation experiments. The key evaluation metrics include effective energy utilization rate, insecticidal operation duration, and energy residual rate. A comparative analysis with the conventional first-half-of-the-night scheme is conducted to comprehensively assess the practical effectiveness of the proposed approach and its potential for pest control applications. Detailed simulation results and corresponding analyses will be presented in subsequent sections.

### 5.1. Simulation Conditions

In the simulation experiments, the SIL utilized ultraviolet light with a wavelength of 365–400 nm, which has been shown to exhibit significant insecticidal efficacy against rice pests [4]. Based on the analysis derived from modeling the phototactic rhythm of rice pests [16], a framework was designed to enable a comprehensive evaluation of the proposed multi-pest energy management algorithm for the SIL-IoT. The simulations were conducted using MATLAB R2019a on a laptop equipped with a 64-bit Windows 10 operating system, 16.0 GB RAM, and a 2.6-GHz Core i7-10750 CPU. To ensure a fair comparison between the proposed method and the conventional first-half-of-the-night switching control scheme for the SIL-IoT, all simulations in this study were performed under unified experimental conditions and parameter settings.

### 5.2. Simulation Results

**Figure 5:**
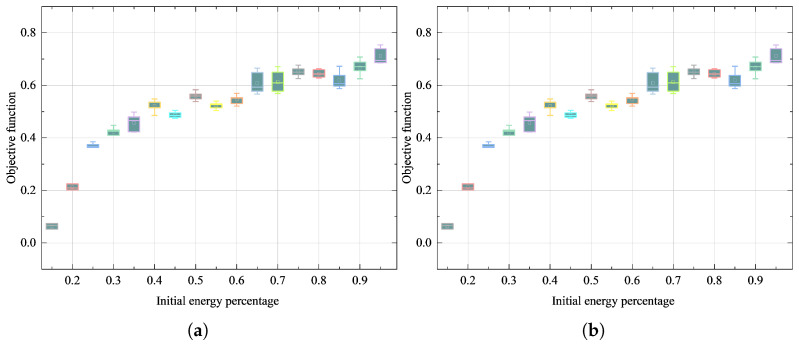
The objective function box diagram of SIL-IoT under different initial energy. (**a**) The objective function box diagram of traditional scheme. (**b**) The objective function box diagram of intelligent scheme.

This work establishes a battery storage capacity partitioning framework based on empirical data from [40] and other literature sources. By monitoring the terminal voltage variations of the battery to indirectly reflect its actual energy state and losses, this approach circumvents the limitations associated with idealized efficiency assumptions. The adopted measurements inherently incorporate actual efficiency losses during battery charge-discharge cycles, thereby ensuring fundamental feasibility of the energy model. Building upon this verified framework, this section systematically validates the feasibility of the proposed scheme through simulation experiments. Based on 30 independent repeated trials, Figure 5 compares the objective function error distributions of the conventional first-half-of-the-night switching control scheme and the dynamic switching control scheme under different initial energy levels using boxplots. The objective function errors across varying initial energy percentages consistently demonstrate high stability, with the error range showing no significant fluctuations as initial energy changes. This characteristic confirms the strong robustness of the objective function to the initial energy parameter.

An improved PSO algorithm with adaptive inertia weight was employed for optimization. Throughout the 30 independent trials, the objective function exhibited low sensitivity to variations in initial energy, further indicating the rationality of the experimental setup and the robustness of the method. These results provide strong evidence that the adopted intelligent PSO algorithm delivers high reliability and stability, regardless of the number of simulation trials. To enhance data reliability, subsequent experiments will use the average values from multiple repeated trials as the basis for simulation results.

This systematic validation process not only ensures the scientific rigor of the experimental conclusions but also establishes a reliable foundation for future research.

Due to the phototactic behavior of pests, significant activity peaks are typically observed during evening, midnight, and early morning hours. Leveraging this characteristic, most commercially available SIL-IoT systems adopt a fixed remote-controlled operational mode from 19:00 to 06:00 [41]. Although pest trap counts are increased by this approach, it is often at the cost of unnecessary energy consumption during periods of low pest activity when the SIL-IoT operates under energy-constrained conditions. This significantly reduces the energy utilization efficiency of the devices [32], especially during continuous cloudy weather or in seasons with limited sunlight.

Field observations have revealed distinct temporal patterns in the nocturnal activity of major rice pests, with peak activity concentrated between 19:00 and 01:00 the next day [13,42]. During this period, insecticidal accounts for 89.13% of the total nightly catch, predominantly consisting of *Cnaphalocrocis medinalis* and *Chilo suppressalis*. After 01:00, the pest population decreases rapidly. Optimizing the operational time window of the SIL-IoT based on this pattern can significantly improve the targeting precision of pest control.

Meanwhile, experimental data indicate that the beneficial-to-harmful insect ratio (i.e., the ratio of beneficial to pest insects) captured by the SIL-IoT is 0.04:1 in vegetable fields [43] and only 0.0089:1 in rice fields [13], both of which are considerably lower than the ratios observed in chemical control practices. Compared with chemical methods (beneficial-to-harmful ratio > 0.15:1), the SIL-IoT reduces the rate of misclassification of natural enemies by more than 65%, effectively contributing to the conservation of farmland biodiversity.

This green pest control technology not only efficiently traps and eliminates pests but also minimizes impacts on non-target insects and natural enemies, thereby helping to maintain ecological balance in agricultural environments. Based on these findings, this paper defines the method where SIL operation is regulated according to pest activity patterns during the first half of the night as the “conventional switching control scheme.” The light activation window is set from 19:00 to 01:00, covering over 85% of peak pest activity. This scheme provides an important reference for optimizing future pest control schemes.

**Figure 6:**
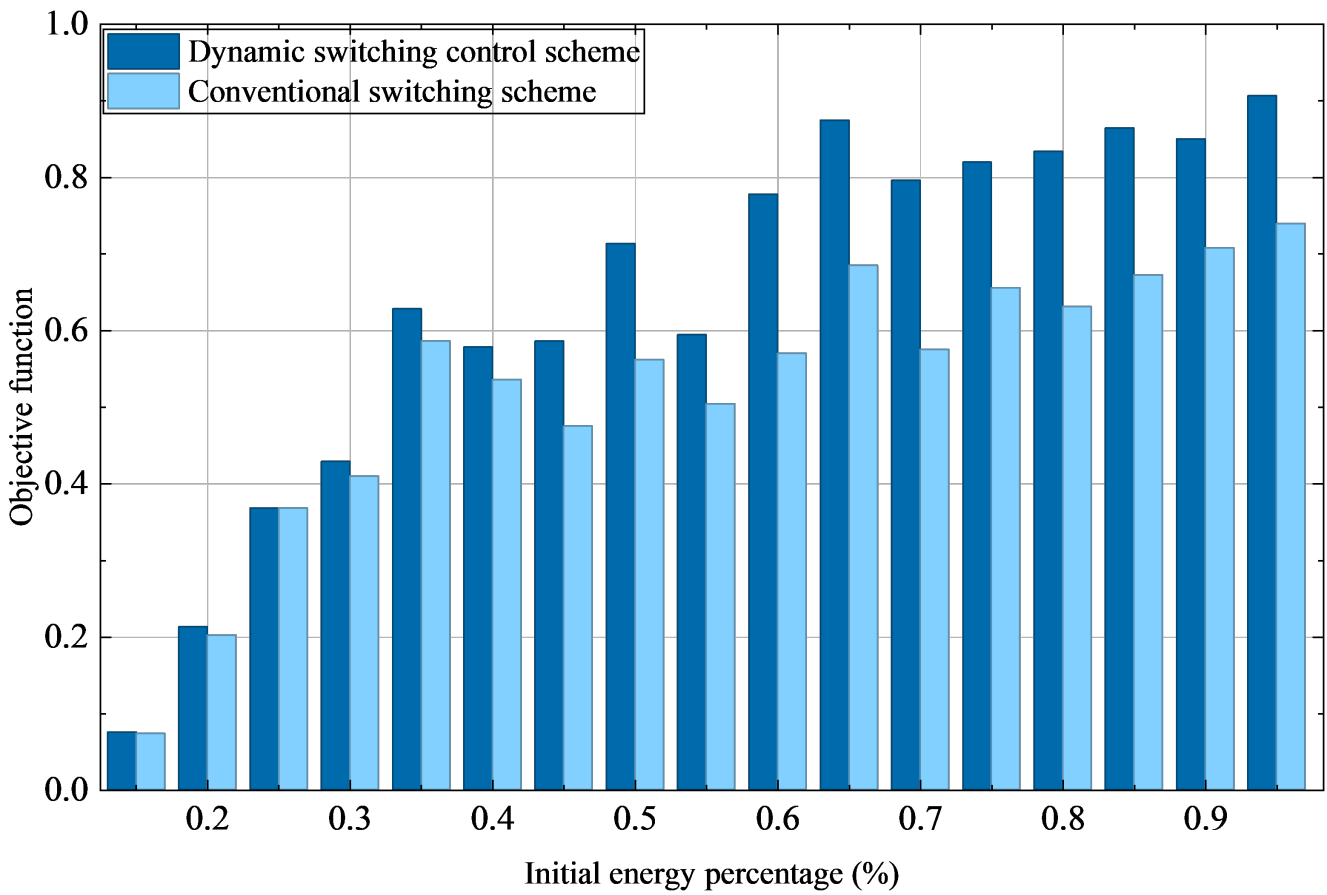
The objective function values of the networked solar insecticidal lamp under different initial energy levels.

Figure 6 illustrates the variation of the objective function values under both conventional and dynamic switching control schemes for networked solar insecticidal lamps at different initial energy levels. The objective function is formulated to simultaneously maximize pest control efficacy and minimize energy consumption, with its value directly reflecting the overall system performance. Experimental results demonstrate that the dynamic switching control scheme consistently achieves higher objective function values than the conventional approach across all initial energy levels, with the performance gap becoming more pronounced at higher energy levels. In contrast, the conventional scheme yields lower objective values and fails to effectively balance energy consumption with operational efficiency, leading to considerable energy waste and compromised control effectiveness. On average, the dynamic scheme improves the objective function value by 11.7% over the conventional method.

The most significant performance gain is observed at 95% initial energy, where the objective value under the dynamic scheme reaches 0.906—an absolute increase of 0.167 (22.6%) compared to 0.739 under the conventional scheme. In the high-energy region (initial energy ≥65%), the dynamic scheme consistently maintains an average objective value above 0.85, substantially outperforming the conventional scheme, which averages around 0.68. Even under energy-constrained conditions, such as at 20% and 30% initial energy, the dynamic approach still achieves performance improvements of 5.6% and 4.7%, respectively, highlighting its adaptability and efficiency. Although both schemes exhibit monotonically increasing objective values with rising initial energy, the dynamic scheme shows a significantly steeper growth rate. These results confirm that the dynamic switching control scheme achieves a better balance between insecticidal efficiency and energy consumption through optimized switching scheduling, especially maintaining stable performance under energy-constrained conditions.

**Figure 7:**
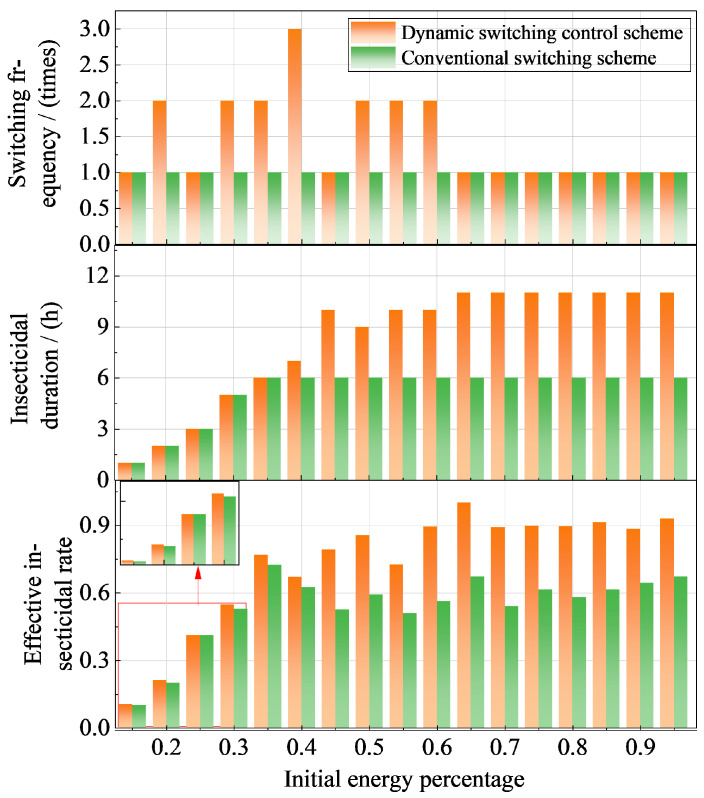
Pest control performance of the networked solar insecticidal lamp under different initial energy levels.

Figure 7 presents a comparative analysis of the performance between the dynamic switching control scheme and the conventional scheme for networked solar insecticidal lamps. Experimental results demonstrate that the dynamic scheme significantly outperforms the conventional one in terms of effective insect elimination, achieving an overall improvement of 17.7% in insecticidal efficacy.

Within the low initial energy range of 15–35%, both schemes exhibit a rapid increase in insect eradication rate as energy levels rise. Although the effective operational duration remains identical, the dynamic scheme achieves higher insect control efficiency through optimized switching frequency. The inset diagram further reveals that under typical low-energy conditions (15%, 20%, 25%, and 30%), the dynamic scheme maintains superior performance, validating its effectiveness and adaptability under energy-constrained scenarios.

The advantage of the dynamic scheme becomes more pronounced in the medium-to-high energy range of 45–70%. At 60% initial energy, the dynamic scheme achieves an insect eradication rate of 90.6%, representing a 30-percentage-point improvement over the conventional scheme’s 60.6%, demonstrating its full potential under adequate energy supply. In contrast, the conventional scheme shows saturated performance improvement beyond 60% initial energy, indicating limitations in energy utilization efficiency. Although the conventional approach exhibits minor fluctuations and gradual improvements with increasing energy, its overall growth remains limited with inferior energy efficiency.

**Figure 8:**
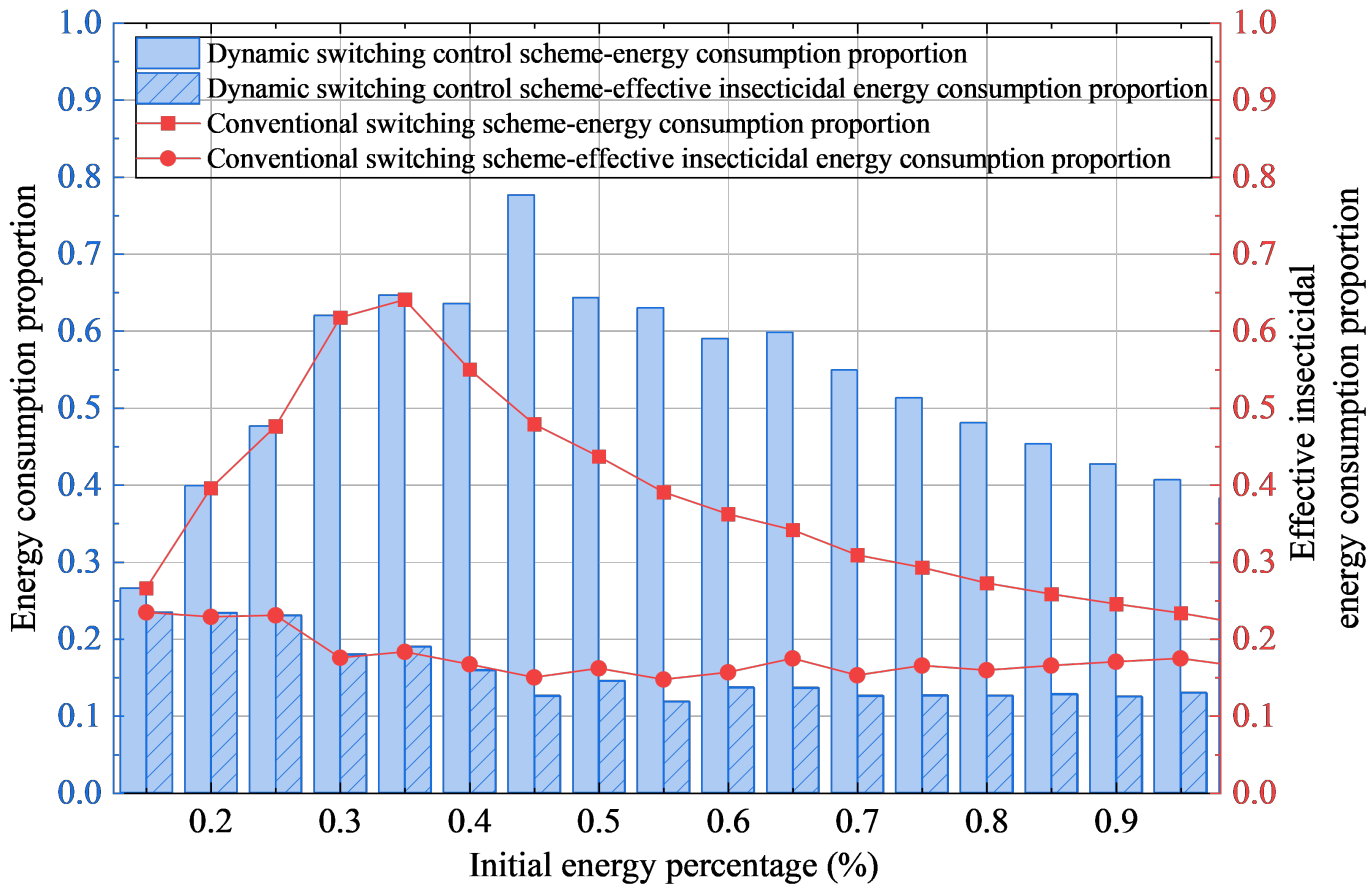
Percentage of energy consumption relative to total energy in networked solar insecticidal lamp under different initial energy levels.

Figure 8 compares the energy utilization efficiency between the dynamic switching control scheme and the conventional scheme under different initial energy conditions. Experimental results indicate that the dynamic scheme demonstrates significant energy optimization capability within the 30–65% initial energy range.

In the low energy range (15–45%), the energy consumption of the dynamic scheme increases with rising initial energy, reaching its peak at 45% while maintaining superior energy utilization efficiency compared to the conventional approach. When the initial energy exceeds 45%, the dynamic scheme exhibits a gradual decline in energy consumption, confirming its effective energy management capability even under high energy conditions. In contrast, the conventional scheme only begins to show a decreasing trend in energy consumption after exceeding 35% initial energy, revealing its limitations in energy regulation flexibility.

Regarding the effective energy allocation for insect elimination, both schemes demonstrate comparable performance within the 15–40% initial energy range. However, when the initial energy exceeds 40%, the actual energy proportion utilized for pest elimination decreases due to reduced nocturnal insect activity, while the energy allocation for attractant light sources increases relatively. Under these conditions, the dynamic scheme achieves higher energy utilization efficiency by effectively utilizing available energy to maintain efficient operation, supported by its enhanced pest elimination performance.

**Figure 9:**
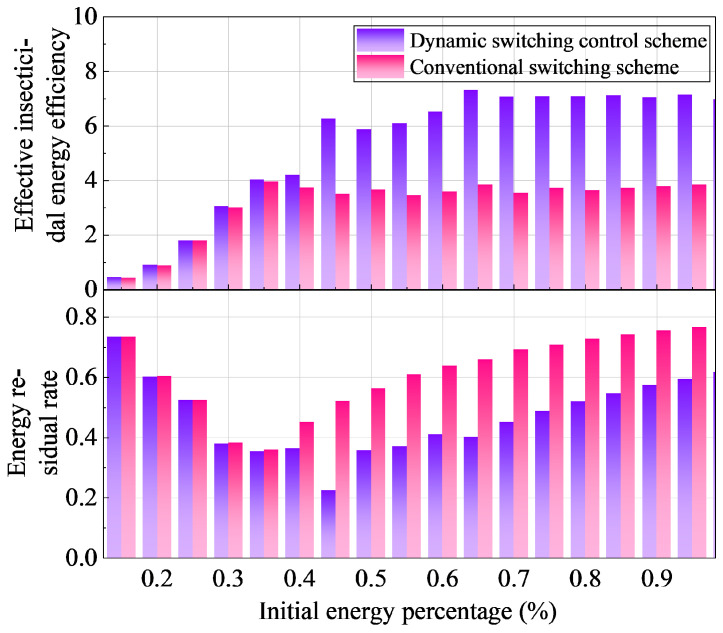
Energy efficiency and remaining energy ratio of networked solar insecticidal lamp under different initial energy levels.

Figure 9 compares the effective insecticidal energy efficiency and energy residual rate between the conventional switching scheme and the dynamic switching control scheme under different initial energy conditions. The effective insecticidal energy efficiency is defined as the ratio of the insect eradication rate to the effective energy utilization rate for insect elimination.

Experimental results demonstrate that as the initial energy level increases, the dynamic switching control scheme exhibits a significant upward trend in effective insecticidal energy efficiency, achieving an average improvement of approximately 66.1% compared to the conventional scheme. Concurrently, due to the increased number of insects attracted, more energy is allocated to actual pest elimination, leading to an approximately 38.5% enhancement in energy utilization efficiency for the dynamic scheme, which is directly reflected in its lower energy residual rate.

When the initial energy exceeds 40%, the dynamic scheme achieves higher pest elimination rates through more frequent switching operations, resulting in increased energy consumption and consequently lower residual energy compared to the conventional scheme, as shown in the figure. Under low initial energy conditions, the performance difference between the two schemes is minimal, with similar energy residual levels, indicating that the dynamic scheme maintains stable performance even under energy-constrained conditions, though with limited improvement in insect attraction efficacy.

In contrast, while the conventional scheme maintains higher residual energy across most energy ranges, this actually reflects its inadequate energy utilization, demonstrating significant energy idling and wastage.

In summary, the dynamic switching control scheme achieves higher energy utilization efficiency while ensuring effective pest elimination through optimized energy allocation and switching strategies, whereas the conventional scheme leads to resource wastage due to insufficient energy utilization efficiency.

## 6. Conclusions and Future Work

### 6.1. Conclusions

This paper systematically addresses the critical issue of energy management in SIL-IoT that neglects the rhythmic behavioral diversity of multiple pests. The limitations of conventional single-pest models are overcome through the first-time integration of nocturnal activity characteristics from major rice pests, including *Cnaphalocrocis medinalis* and *Chilo suppressalis*. An optimized dynamic switching control and energy management scheme is subsequently proposed based on a hybrid model of pest phototactic rhythm. By comprehensively considering energy consumption, residual energy, and energy cost, the proposed scheme was validated through simulations of an intelligent PSO-based switching control scheme, demonstrating significant performance improvements and excellent robustness under various initial energy conditions.

Simulation results demonstrate that, compared with the conventional switching control scheme, the dynamic switching control scheme achieves the 17.7% improvement in the average insecticidal rate, increases the effective insecticidal energy efficiency by approximately 66.1%, and enhances the energy utilization rate by about 38.5%. The proposed scheme not only significantly improves the precision of pest control and energy utilization efficiency, but also promotes the effective application of networked solar insecticidal lamps in smart agriculture. This research provides theoretical insights and practical guidance for advancing intelligent pest control in complex agricultural environments, supporting more precise and sustainable pest management.

### 6.2. Future Work

Building upon this paper, future work will systematically focus on the following aspects of exploration and application advancement:

- Enhanced Environmental Adaptability and Model Optimization. Further investigation will be conducted into the distribution patterns and behavioral characteristics of pest populations under diverse meteorological conditions, establishing a dynamic prediction model that integrates environmental factors. Concurrently, through field experiments employing multi-wavelength light sources, we will systematically analyze the attraction efficiency of specific spectral bands on target pests, thereby constructing an intelligent control mechanism based on spectral-environment-pest behavior correlation analysis. On this basis, we will further optimize model complexity and strategy feasibility to reduce system deployment and maintenance costs while maintaining control accuracy, significantly enhancing the environmental adaptability, eco-friendliness, and promotion value of the technical solution.
- Battery System Performance Optimization. This research will conduct an in-depth investigation into the energy conversion efficiency of solar cells under various environmental conditions, with a focus on the impacts of temperature, irradiance intensity, and battery aging on charge-discharge performance. By establishing a battery degradation model and lifetime prediction method, the energy management strategy will be optimized to enhance the long-term operational energy utilization efficiency and reliability of the system.
- System Extension and Multi-Objective Coordination. The current framework will be extended to other crop-pest systems to examine its applicability and scalability across different ecological regions and agricultural scenarios. Multiple objective constraints—such as energy consumption, residual energy rate, and energy cost—will be incorporated into the research and design to achieve holistic optimization of pest control effectiveness and energy utilization efficiency.

The advancement of these research directions will not only improve the insecticidal performance and energy utilization efficiency of SIL but also provide key technological support for the intelligent and sustainable development of green agriculture.

## Figures and Tables

**Table 1:** Research focus and impact on SIL-IoT dynamic switching control and intelligent energy management.

Papers	Research Direction	Characteristics	Impact on Dynamic Switching Control and Intelligent Energy Management
[17]	Node Deployment Strategy	Minimizes SIL deployment quantity while ensuring area coverage, reducing redundancy	Deployment location directly affects solar energy reception efficiency, influencing energy collection and storage
[18]	Fault Diagnosis Technology	Classifies faults using sensor historical data for rapid identification and resolution	Efficient fault diagnosis reduces energy waste and prevents losses due to equipment malfunctions
[19,20]	Electromagnetic Interference Resistance	IoT modules within 25 cm of the insecticidal lamp’s high-voltage metal mesh are prone to data collection interference	Anti-interference measures ensure stable operation, minimizing energy loss from faults or interference
[21,22]	Pest Monitoring and Localization Technology	Utilizes multiple sensors for accurate pest counting and hotspot area identification	Precise pest monitoring optimizes operational timing, reducing unnecessary energy consumption
[23]	Equipment Maintenance Solution	Remote maintenance system enables task viewing and allocation when equipment is damaged	Regular maintenance ensures long-term efficient operation, lowering costs and reducing energy usage

**Table 2:** Comparison of different intelligent switch schemes.

Papers	Method/Technique	Objective/Outcome	Adjustment Mechanism	Energy Source	Application Scenario
[24]	Machine learning algorithm	Optimize water and fertilizer use, maximize crop yield	Adjust motor start/stop time precisely	Solar energy	Farmland
[25]	Neural network	Achieve efficient irrigation	Adjust irrigation switching times	N/A	Greenhouses and farms
[26]	Fuzzy logic	Prevent motor burnout due to water level fluctuations	Adjust water pump switching times	Solar energy	Farmland
[27]	Fuzzy control algorithm	Optimize plant growth, reduce water and energy use	Control irrigation valve switching	N/A	Tomato cultivation
[28]	Intelligent decision algorithm	Reduce irrigation water use, improve water productivity	Schedule irrigation times for corn fields	N/A	Cornfields
[29]	Fuzzy algorithm	Intelligent and precise irrigation system	Adjust irrigation time for apple trees	Solar energy	Apple orchards
[30]	Power management and control	Save energy and enable autonomous environmental monitoring	Adjust power switching automatically	Solar energy	Botanical gardens
[31]	Power management system	Enable automated control with sensors and solar panels	Adjust critical environmental parameters	Solar and wind energy	Greenhouses
[32]	Genetic algorithm	Optimize pest control efficiency of solar lamps	Schedule pest control operation periods	Solar panels and batteries	Farmland
Our paper (2025)	Particle swarm optimization (PSO)	Improve both pest control effectiveness and energy usage	Adjust pest control operation periods	Solar panels and batteries	Farmland

## Data Availability

The original contributions presented in this study are included in the article. Further inquiries can be directed to the corresponding author.

## Nomenclature

$t_{m}$	time slots; per 60 min is a pest recording duration 0<t≤11
$p_{1}\left(t_{m}\right)$	the phototactic rhythm of *Cnaphalocrocis medinalis*
$p_{2}\left(t_{m}\right)$	the phototactic rhythm of *Chilo suppressalis*
$p_{static}\left(t_{m}\right)$	the time dependent one-dimensional phototactic rhythm static model
$f(t_{m})$	the dynamic phototactic rhythm of these two major rice pests
$F(t_{m})$	the fitness function
$\xi \left(t_{m}\right)$	the Gaussian white noise
σ	the standard deviation
$\alpha_{t_{m}}$	the switching behavior over discretized time intervals
$T_{on}$	the total insecticidal operation time
$T_{off}$	the trap pest light source termed the non-operational period
$E_{0}$	the initial remaining remaining energy of the SIL storage battery in the daytime
$E_{c}^{t_{m}}$	the energy consumed at night time
$E_{lc}^{t_{m}}$	the energy consumption of the lamp
$E_{mc}^{t_{m}}$	the energy consumption of the metal mesh
$E_{pc}^{t_{m}}$	the energy consumption of the insecticidal
$E_{tc}^{t_{m}}$	the energy consumption of the IoT communication transmission
$E_{a}$	the total capacity of the 48V lead-acid battery
$E_{r}^{t_{m}}$	the residual energy of the SIL
$SOC_{0}$	the energy state in the SIL’s 48V lead-acid battery from the moment the lamp is turned on at night time
$SOC_{t_{m}}$	the energy state of the SIL at the $t_{m}$ time slot
$W^{t_{m}}$	the comprehensive benefit model
γ,β	the weighting coefficients
$X_{id}$	the candidate solution
*i*	the particle
*d*	dimension
k+1	the generation
ω	the inertia weight
$V_{id}$	the direction and step size of the search
$c_{1},c_{2}$	the learning factors
$r_{1},r_{2}$	the random numbers uniformly distributed
$g_{\mathrm{best}\;,d}$	the historical best position of the swarm in dimension *d* (global best)
$x_{id}^{k}$	the position of particle *i* in dimension *d* at iteration *k*
$x_{id}^{k}+1$	the particle *i* in dimension *d* at iteration k+1

## References

[1] Ahouandjinou A.S.R.M., Kiki P.M.A.F., Assogba K. Smart environment monitoring system by using sensors ultrasonic detection of farm pests. Proceedings of the 2017 2nd International Conference on Bio-engineering for Smart Technologies (BioSMART).

[2] Wang Z., Qiao X., Wang Y., Yu H., Mu C. (2024). IoT-based system of prevention and control for crop diseases and insect pests. Front. Plant Sci..

[3] Liu J., Cheng J., He C., Gao C. (2024). Advancements on the insecticides application and key technique in the control of disaster pests in paddy field. Mod. Agrochem..

[4] Yao H., Shu L., Yang F., Jin Y., Yang Y. (2022). The phototactic rhythm of pests for the Solar Insecticidal Lamp: A review. Front. Plant Sci..

[5] Ilahi N.A., Musyafiq A.A., Pradana M.F., Alimudin E., Fadlilah I., Husna K.S., Nagara E.S., Santoso A. (2025). IoT-Enabled Solar-Powered Pest Control for Rice Agriculture: Monitoring and Efficiency of Light-Based Traps. J. Power Energy Control..

[6] Frank E.G. (2024). The economic impacts of ecosystem disruptions: Costs from substituting biological pest control. Science.

[7] Kim K., Huang Q., Lei C. (2019). Advances in insect phototaxis and application to pest management: A review. Pest Manag. Sci..

[8] Asna F., Nizam Y. (2024). Designing a solar powered light trap to control pest moths in Hulumale’urban farming field. AIP Conf. Proc..

[9] Khan A., Hasan W., Bisht K., Khan R.M., Chattopadhyay D., Majumder J., Khan I., Rabeek S.M., Ahmad S. (2024). Insect Phototaxis Mechanisms Innovations in Pest Control Strategies and Applications. Uttar Pradesh J. Zool..

[10] Park J., Lee H. (2017). Phototactic behavioral response of agricultural insects and stored-product insects to light-emitting diodes (LEDs). Appl. Biol. Chem..

[11] Wang K., Gao Q., Li L., Liu W., Lei C., Wang X. (2020). Current development status of agricultural insect-pest light trap in China. Insect Res. Cent. China.

[12] Sun X., Tang L., Qian S., Shen X. (2012). Experimental Study on Solar-Powered Intelligent Insecticidal Lamps in Paddy Fields. J. Green Sci. Technol..

[13] Tu H., Tang N., Kang N., Zhou J., Lin A. (2016). LED multispectral circulation solar insecticidal lamp application in rice field. Trans. Chin. Soc. Agric. Eng..

[14] Pan H., Liang G., Lu Y. (2021). Response of different insect groups to various wavelengths of light under field conditions. Insects.

[15] Yang X., Lu Y., Liang G. (2020). Insect phototaxis behavior and light trapping technology. Illum. Eng. J..

[16] Yao H., Shu L., Lin W., Huang K., Martínez-García M., Zou X. (2024). Pests phototactic rhythm driven solar insecticidal lamp device evolution: Mathematical model preliminary result and future directions. IEEE Open J. Ind. Electron. Soc..

[17] Yang F., Shu L., Huang K., Li K., Han G., Liu Y. (2020). A Partition-Based Node Deployment Strategy in Solar Insecticidal Lamps Internet of Things. IEEE Internet Things J..

[18] Yang X., Shu L., Li K., Nurellari E., Huo Z., Zhang Y. (2023). A Lightweight Fault-Detection Scheme for Resource-Constrained Solar Insecticidal Lamp IoTs. Sensors.

[19] Huang K., Li K., Shu L., Yang X., Gordon T., Wang X. (2020). High voltage discharge exhibits severe effect on ZigBee-based device in solar insecticidal lamps internet of things. IEEE Wirel. Commun..

[20] Chen M., Liu Y., Shu L., Li K., Yang X., Yang F. SILGAN: Generative Adversarial Networks for Multimedia Data Compression in Solar Insecticidal Lamps Internet of Things. Proceedings of the IECON 2023-49th Annual Conference of the IEEE Industrial Electronics Society.

[21] Gao L. (2018). Design of Orchard Pest Information Monitoring System Based on IoT Technology. Ph.D. Thesis.

[22] Jiang Z., Shu L., Yang X., Huang K., Yao H., Su Q. (2024). An Insecticidal Counting Method Based on Discharge Sound and Discharge Voltage of Solar Insecticidal Lamp. IEEE Trans. Consum. Electron..

[23] Ma Q., Tian M., Tang W. (2017). Research and Design of a WSN-Based Distributed Remote Control System for Solar Insecticidal Lamps. Internet Things Technol..

[24] Rao R.N., Sridhar B. IoT based smart crop-field monitoring and automation irrigation system. Proceedings of the 2018 2nd International Conference on Inventive Systems and Control (ICISC).

[25] Nawandar N.K., Satpute V.R. (2019). IoT based low cost and intelligent module for smart irrigation system. Comput. Electron. Agric..

[26] AlAli A., Al Nabulsi A., Mukhopadhyay S., Awal M.S., Fernandes S., Ailabouni K. (2019). IoT-solar energy powered smart farm irrigation system. J. Electron. Sci. Technol..

[27] Benyezza H., Bouhedda M., Rebouh S. (2021). Zoning irrigation smart system based on fuzzy control technology and IoT for water and energy saving. J. Clean. Prod..

[28] Sharifnasab H., Mahrokh A., Dehghanisanij H., Łazuka E., Łagód G., Karami H. (2023). Evaluating the use of intelligent irrigation systems based on the IoT in grain corn irrigation. Water.

[29] Benzaouia M., Hajji B., Mellit A., Rabhi A. (2023). Fuzzy-IoT smart irrigation system for precision scheduling and monitoring. Comput. Electron. Agric..

[30] Balle M., Xu W., Darras K.F., Wanger T.C. (2024). A power management and control system for environmental monitoring devices. IEEE Trans. Agrifood Electron..

[31] Chen K.C., Chiu M.C., Cheng H.C., Wang Y.H., Lan T.S. (2025). Greenhouse for Effective Agriculture with Electricity Generation and Water Collection Systems. Sensors Mater..

[32] Yao H., Shu L., Yang Y., Martínez-García M., Lin W. (2025). Silic: Intelligent on/off control for networked solar insecticidal lamps. IEEE/CAA J. Autom. Sin..

[33] Guo X., Shu L., Yang X., Nurellari E., Li K., Du B., Yao H. (2021). Two-hop energy consumption balanced routing algorithm for solar insecticidal lamp Internet of Things. Sensors.

[34] Zhang Z. (2013). Monitoring and Population Dynamics Analysis of Important Migratory Pest Insects in Northern China. Ph.D. Thesis.

[35] Yang H. (2014). Study on Light-Trapped Behavior of *Sogatellla furcifera* (Horvath) and *Nilaparvata lugens* (Stal). Ph.D. Thesis.

[36] Zhang X., Jia Y., Wen Y., Zhang Y., Wan G., Chen F. (2017). Behavioral rhythms of three lepidopteran pests; *Mythimna separata*, *Agrotis ypsilon* and *Helicoverpa armigera*. Chin. J. Appl. Entomol..

[37] Gu G., Ge H., Cheng X., Han J., Yin J. (2004). Study on and application of the rhythm of several niht-active insects to light trap in the night. J. Hubei Agric. Coll..

[38] Zhu S., Liu B., Liu M., Qi H. (2025). Stability analysis of a stochastic discrete pest-natural enemy model with integrated pest management strategy. Stochastics Dyn..

[39] Lin S. (2016). The Research and the Manufacture of Solar LED Insecticidal Light. Ph.D. Thesis.

[40] Chen G. (2011). Design and Implementation of a Solar-Powered Optically Controlled High-Voltage Triple-Wave Pest Attraction Lamp. Ph.D. Thesis.

[41] Zhang T., Huang P. (2005). The trapping effect of Jiaduo Frequoscillation pest killing lamp on rice-stem borer. China Agric. Technol. Ext..

[42] Yang J., Liang C., Shen B., Zhang Q., Tian C., Huang Y. (2012). Study on rhythm of insect flapping lamp. J. Anhui Agric. Sci..

[43] Liu L., Wang F., Chen C., Guan Y., Liang J., Liu Z. (2023). Research on the Application Effect of Intelligent Wind-Suction Solar Insecticidal Lamps on Vegetables. Mod. Agric. Sci. Technol..

